# Anti-inflammatory effects of the gorgonian *Pseudopterogorgia elisabethae *collected at the Islands of Providencia and San Andrés (SW Caribbean)

**DOI:** 10.1186/1476-9255-6-5

**Published:** 2009-03-10

**Authors:** Hebelin Correa, Alba Lucia Valenzuela, Luis Fernando Ospina, Carmenza Duque

**Affiliations:** 1 Departamento de Química, Universidad Nacional de Colombia, Cra. 30 N° 45 -03, Bogotá D.C, Colombia; 2 Departamento de Farmacia, Universidad Nacional de Colombia, Cra. 30 N° 45 -03, Bogotá D.C, Colombia

## Abstract

**Background:**

We are reporting for the first time the *in vivo *anti-inflammatory activity of extracts and fractions, and *in vitro *anti-inflammatory activity of pure compounds, all isolated from *Pseudopterogorgia elisabethae *collected at the Providencia (chemotype 1) and San Andrés (chemotype 2) Islands (SW Caribbean).

**Methods:**

Extracts from *P. elisabethae *were fractionated on silica gel to yield fractions: F-1 (pseudopterosins PsQ, PsS and PsU) and F-2 (amphilectosins A and B, PsG, PsK, PsP and PsT and *seco*-pseudopterosins *seco*-PsJ and *seco*-PsK) from chemotype 1, and F-3 (elisabethatrienol, 10-acetoxy-9-hydroxy- and 9-acetoxy-10-hydroxy-amphilecta-8,10,12,14-tetraenes (interconverting mixture) and amphilecta-8(13),11,14-triene-9,10-dione) from chemotype 2. By using preparative RP-HPLC and spectroscopic means, we obtained the pure PsG, PsK, PsP, PsQ, PsS, PsT, PsU, *seco*-PsK and the interconverting mixture of non-glycosylated diterpenes (IMNGD). The anti-inflammatory properties of extracts and fractions were evaluated using *in vivo *model "12-*O*-tetradecanoyl-phorbol-acetate (TPA)-induced mouse ear oedema". The activities of pure compounds and of the IMNGD were evaluated using *in vitro *assays myeloperoxidase (MPO) release (by human polymorphonuclear neutrophils (PMNs)), nitric oxide release (by J-774 cells) and scavenger activity on NO.

**Results:**

In the *in vivo *anti-inflammatory assay, extracts and F-3 showed low inhibition levels of inflammation compared to indomethacin, F-1 and F-2. Additionally, we evaluated the MPO release to the inflammation site, and found a marked inhibition of MPO levels by all extracts and fractions, even superior to the inhibition shown by indomethacin.

Furthermore, in the MPO *in vitro *assay, IMNGD, PsQ, PsS, PsT and PsU, exhibited higher inhibition levels compared to dexamethasone and indomethacin. In the NO release *in vitro*, IMNGD, PsP and PsT were the most potent treatments. Finally, because the PsG, PsP and *seco*-PsK did not exhibit any NO scavenger activity, they should inhibit the inducible Nitric Oxide Synthase (iNOS) or other routes that influence this enzyme. Alternatively, PsQ, PsS, and PsU did show scavenger activity.

**Conclusion:**

All results presented contribute to demonstrate that the compounds isolated in this work from *P. elisabethae *are promising molecules with an interesting anti-inflammatory activity profile. Additionally, the results obtained could provide preliminary insights towards their structure-activity relationship.

## Background

The pseudopterosins and *seco*-pseudopterosins, diterpene glycosides isolated from the Caribbean gorgonian octocoral *Pseudopterogorgia elisabethae*, have shown potent anti-inflammatory and analgesic properties through *in vitro *and *in vivo *assays [1-7], usually involving versatile modes of action [4,8]. They have a particular interest due to their superior anti-inflammatory properties compared to the commercial drug indomethacin. Furthermore, these compounds appear to inhibit eicosanoid biosynthesis by inhibiting phospholipase A2 (PLA2), 5-lipoxygenase (5-LO) and cycloxygenase (COX), degranulation of leukocytes and the consequent liberation of lisosomal enzymes [3,4].

At present, partially purified extracts from *P. elisabethae *collected in the Bahama, rich in pseudopterosins are currently incorporated into several skin care preparations marketed by Estée Lauder, due to their excellent anti-inflammatory and analgesic properties [9].

Likewise, the pseudopterosins A-D have been licensed to OsteoArthritis Sciences Inc., for medical use as anti-inflammatory drugs. This pharmaceutical company has completed preclinical tests and developed a potent derivative of PsA called methopterosin (OAS1000), which is in clinical phase I/II trial as a wound healing and anti-inflammatory agent [10,11].

The high degree of chemical variation between different specimens of *P. elisabethae *collected at various sites in the Caribbean region has been acknowledged by several authors. So far, 17 pseudopterosins (PsA-PsO, PsX and PsY) isolated from specimens collected in the Bahamas [1,12,13], Bermuda [12], and the Florida Keys [5] have been reported. The structurally related *seco*-pseudopterosins A-D have also been identified in *Pseudopterogorgia kallos *collected near the Marquesas Keys in Florida [2], the *seco*-PsE-G and *seco*-PsJ isolated from *P. elisabethae *collected at the Long Key, Florida [2,14], and *seco*-PsH-I isolated from *P. elisabethae *collected at the San Andrés Island [6].

Recently, as a part of our continuous search for biologically active compounds from marine organisms, we evaluated the extracts from *P. elisabethae *collected at Providencia and San Andrés Islands (SW Caribbean) by LC-MS, and found two distinct chemotypes that were characterized based on their pseudopterosin and related compound compositions. This correlated well with the geographical distribution [15]. Chemotype 1, found almost exclusively in Providencia Island, was mainly characterized by the presence of PsP-PsV, PsG and PsK, amphilectosins A and B, and two *seco*-pseudopterosins (*seco-*PsJ and *seco*-PsK) [16,17]. Chemotype 2, found in San Andrés Island, was revealed to contain several non-glycosylated diterpenes such as an elisabethatriene analog named by us as elisabethatrienol, 10-acetoxy-9-hydroxy- and 9-acetoxy-10-hydroxy-amphilecta-8,10,12,14-tetraenes (isolated as an interconverting mixture (IMNGD)) and amphilecta-8(13),11,14-triene-9,10-dione, along with smaller amounts of pseudopterosins [14,17]. As far as we know, there is only one work reporting the anti-inflammatory activity of the PsQ as an inhibitor of both superoxide anion (O_2_^-^) and thromboxane B_2 _(TXB_2_) both produced by activated rat neonatal microglia *in vitro *[6]. So far still nothing has been published on the activity of the other diterpenes isolated from specimens collected at Providencia and San Andrés Islands.

**Figure 1:**
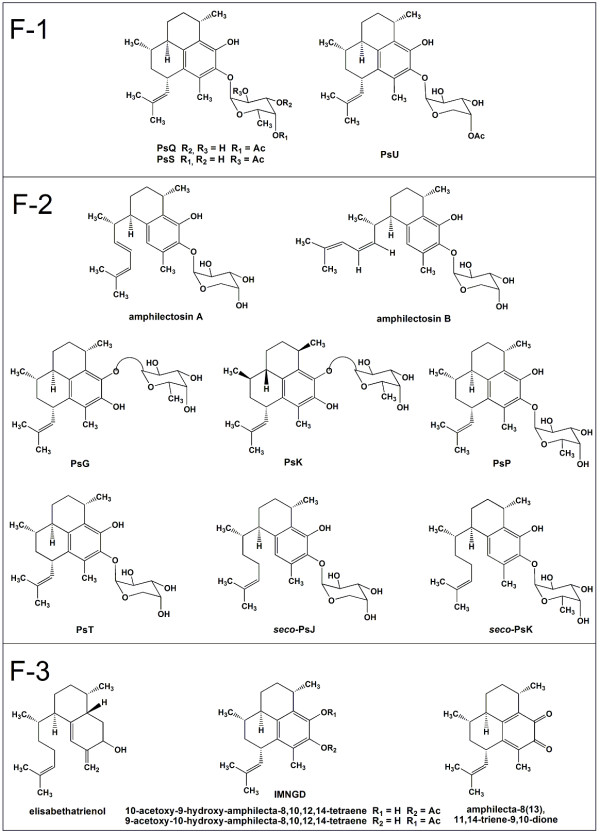
**Chemical structures of compounds isolated from *P. elisabethae***. F-1 and F-2: diterpenes isolated from chemotype 1. F-3: diterpenes isolated from chemotype 2.

We evaluated for the first time the anti-inflammatory activity of pseudopterosins, *seco*-pseudopterosins and the related IMNGD (Figure 1) isolated from the two chemotypes of *P. elisabethae *collected at the Providencia and San Andrés Islands (SW Caribbean) [16,17]. The extracts and fractions from the two chemotypes were assayed using the *in vivo *model "12-*O*-tetradecanoyl-phorbol-acetate (TPA)-induced mouse ear oedema" [18]. Compounds PsG, PsK, PsP, PsQ, PsS, PsT, PsU, *seco*-PsK and IMNGD were evaluated using *in vitro *anti-inflammatory screenings as myeloperoxidase (MPO) assay (released by human polymorphonuclear neutrophils (PMNs)) [19,20], nitric oxide release (cell based assay) and scavenger activity on this radical [21].

## Methods

### Extraction of coral material and isolation of compounds

Fragments of individual colonies of *P. elisabethae *were collected by SCUBA (Ca. 20–30 m depth) at different sites of Providencia and San Andrés Islands (SW Caribbean). Corals were identified by Dr. M. Puyana and vouchers specimens deposited at the invertebrate collection of Museo de Historia Natural Marina Colombiana (MHNMC) at Instituto de Investigaciones Marinas de Punta Betín (INVEMAR), coded as INV CNI 1612–1616. The dried colony fragments (30 g) from each location were extracted separately with dichloromethane-methanol (1:1) mixture. Resultant extracts were filtered and concentrated by rotary evaporation to obtain dark green oily extracts. The extracts were classified by their LC-MS profile as chemotype 1 (Providencia extract) and chemotype 2 (San Andrés extract), according to our previous report [15]. Each extract was subjected separately to silica gel column chromatography and eluted with 500 ml of each solvent mixture of increasing polarity (hexane-diethyl ether 1:1, 2:8; diethyl ether; diethyl ether-ethyl acetate 8:2, 1:1, 2:8; ethyl acetate; ethyl acetate-ethanol 8:2, 1:1; 2:8 and ethanol) as previously described [16,17]. Thus, we obtained fraction 1 (F-1) (eluted with diethyl ether-ethyl acetate 8:2) containing PsQ (47.5%), PsS (7.0%), and PsU (44.2%), and fraction 2 (F-2) (eluted with diethyl ether-ethyl acetate 2:8) containing amphilectosins A (4.8%) and B(4.9%), PsG (28.3%), PsK (13.3%), PsP (19.7%) and PsT (11.6%) and *seco*-PsJ (8.1%) and *seco*-PsK (6.7%) from chemotype 1; the fraction 3 (F-3) (eluted with hexane-diethyl ether 2:8) containing elisabethatrienol (6.7%), IMNGD (1:1) (84.5%), amphilecta-8(13),11,14-triene-9,10-dione (2.8%) and other minor diterpenes (6.0%) were obtained from chemotype 2. Final purification of all compounds was performed on RP-HPLC, using MeOH-water (9:1) as mobile phase with a 1.0 ml/min flow rate. The isolated compounds were carefully identified by spectrospic means according to the procedure described in our earlier publications [16,17] and their purity checked by HPLC and ^13^C NMR including DEPT.

### Laboratory animals

Eight to ten week old ICR mice (35–42 g) of both sexes were purchased from the animal center at the Departamento de Farmacia, Universidad Nacional de Colombia. All mice were acclimatized under standard laboratory conditions, kept alternatively at 12 h of light and darkness and fed with food and water *ad libitum*. Room temperature was maintained at 20 ± 2°C. Animal experiments were carried out in accordance with the criteria outlined in "Guide for the Care and Use of Laboratory Animals" [22] approved by the local Animal Ethical Committee and the guide 008430 of 1993 issued by the Health Department of Colombia [23].

### Drugs

The following substances were purchased from Sigma (St Louis, USA): 12-*O*-tetradecanoyl-phorbol-acetate (TPA), indomethacin, dexamethasone, L-NIO, curcumin, hexadecyltrimethylammonium bromide (HTAB), tetramethylbencidine, hydrogen peroxide, formaldehyde, dimethylformamide, sodium nitroprusside, sodium acetate, sulfanilamide, lipopolysaccharide (LPS) from *Escherichia coli*, Hanks' balanced salt solution (HBSS), Dulbecco's modified Eagle's medium (DMEM), phosphate-buffered saline (PBS), fetal bovine serum (FBS), gentamycin and calcium ionophore A23187. Ficoll-Paque was purchased from ICN (USA) and organic solvents from Merck Co. (Germany).

### Cell culture and cell line J-774 preparation

The J774 murine macrophage cell line was maintained as an adherent culture and was grown as a monolayer in a humidified incubator (95% air; 5% CO_2_) at 37°C in 75 cm^2 ^flasks containing DMEM supplemented with 10% (v/v) FBS, and 50 μg/ml gentamycin [24]. The cells were detached mechanically and viability was evaluated by trypan blue exclusion assay.

### Human polymorphonuclear neutrophils preparation

A sample of cells were obtained from the peripheral blood of healthy subjects, and PMNs were extracted employing the standard techniques of dextran sedimentation, centrifugation on Ficoll-Paque (1.077 g/ml), and hypotonic lysis of contaminating red blood cells. The cells were washed twice and resuspended in HBSS and used immediately. The PMNs purity was 98–100% (Turk exclusion test) and viability ≥ 99%, as determined by the trypan blue exclusion assay. All donors were non-smokers and none had received medication for a period of 3 days prior to donation. Informed consent was obtained from all participants.

### Topical anti-inflammatory activity

Topical anti-inflammatory activity of the extracts and fractions of the two chemotypes was studied using the method described by De Young [18]. Ten ICR mice were used for each treatment group. Oedema was induced on the right ear by topical application of 2.5 μg/ear of TPA in acetone. The left ear was left untreated and used as control. Extracts, fractions and indomethacin (0.5 mg/ear) were dissolved in acetone and applied to right ear simultaneously with TPA. Four hours after the inflammation induction the animals were sacrificed and a biopsy (6 mm diameter) of both ears (left and right) was performed. The oedema was measured as an increase in ear thickness due to the TPA agent application by difference in weight between both ears. The inflammation inhibition percentage was evaluated as the weight difference between treated and non- treated ears of each animal compared to the control group (vehicle).

### Myeloperoxidase assay in mouse ear oedema tissues

Ear sections of each treatment were placed in 1 ml of PBS pH 6.5 containing 0.5% HTAB and homogenized (45 s at 0°C) in a homogenizer (POLYTRON). The homogenate was decanted in a microfuge tube and centrifuged at 1250 rpm at 4°C for 15 min. Triplicate 25 μl samples of the resulting supernatant were added to 96 well microtitre plates. For the assay, 125 μl of HBSS pH 7.4, 50 μl of PBS pH 5.4 and 20 μl of 0.012% hydrogen peroxide were added to the wells and the plates were incubated at 37°C for 5 min. The reaction was started by adding 20 μl of 18 mM tetramethylbencidine in 8% aqueous dimethylformamide. Plates were incubated at 37°C for 3 min and then the reaction was stopped by adding 30 μl of 1.5 M sodium acetate, pH 3.0 [25]. Enzyme activity was determined colorimetrically using a BIORAD 550 microplate reader set to measure absorbance at 620 nm and expressed as the inhibition percentage of MPO levels determined as the absorbance difference between the control group (vehicle) and the treated group compared to the absorbance observed in the control.

### Myeloperoxidase assay in human polymorphonuclear neutrophils

This assay was performed as described previously by Bradley et al. [25]. PMNs (2.5 × 10^6 ^cells/well) were suspended in HBSS. Cell viability (>97%) was determined with the 3- [4,5-dimethylthiazol-2-yl]-2,5-diphenyl-tetrazolium bromide (MTT) cytotoxicity assay. IMNGD (50 μg/ml), pure compounds (10 μM), indomethacin (10 μM) and dexamethasone (10 μM), were added to cells and the mixture incubated at 37°C for 5 min. Subsequently, the cells were activated by calcium ionophore A23187 (1 μM) and incubated at 37°C for an additional 10 min. The reaction was stopped by centrifugation (3000 rpm, 4°C and 15 min) and the enzyme activity in the supernatant was determined as described above for MPO assay in mouse ear oedema tissues.

### Effect on NO production in J774 macrophages

Assay was carried out as described by CYTED [26]. The J774 cells were plated in 96 well culture plates at a density of 2.0 × 10^5 ^cells/well and allowed to adhere for 2 h in DMEM supplemented with 5% FBS and gentamycin (50 μg/ml), and cultured at 37°C in humified 95% air, 5% CO_2_. Thereafter the medium was replaced with fresh medium and cells were activated by LPS (100 μg/ml) from *E. coli*. Thirty minutes before LPS test IMNGD, pure compounds, dexamethasone and L-NIO were added to cells at various concentrations. After 18–20 h culture medium was removed, centrifuged and the supernatant was used for the determination of nitrite (NO_2_^-^) production. Cell viability (>95%) was determined with the Alamar blue assay. NO_2_^- ^levels in culture media from J774 macrophages were measured 24 h after LPS or compound challenge with the Griess reaction [26]. After 5 min incubation at room temperature the absorbance in the plate was measured at 570 nm using a BIORAD 550 microplate reader set. The results were expressed as the inhibition percentage of NO measuring the absorbance difference between absorbance of maximum levels of NO_2_^- ^(LPS stimulated cells) compared to the absorbance of each treatment.

### Nitric oxide scavenger assay

Sodium nitroprusside in aqueous solution at physiological pH spontaneously generates nitric oxide, which interacts with oxygen to produce nitrite ions these can be estimated by the use of Griess reagent as described previously by Marcocci et al. [27]. Scavengers of nitric oxide compete with oxygen leading to reduced production of nitric oxide. Sodium nitroprusside (5 μM) in PBS was mixed with different concentrations of the IMNGD and pure compounds dissolved in methanol and incubated at 25°C for 120 min. The samples were then reacted with Griess reagent. The absorbance of the chromophore formed during the diazotization of nitrite with naphthylethylenediamine was measured at 570 nm using a BIORAD 550 microplate reader set and referred to the absorbance of standard solutions of potassium nitrite treated in the same way with Griess reagent. Curcumin was used as positive control. The results were expressed as scavenger percentage of NO, measuring the absorbance difference between absorbance of maximum levels of NO compared to the absorbance of each treatment.

### Statistical analysis

Results are presented as mean ± standard error of mean (S.E.M.). Data was subjected to descriptive statistics and analysis of variance (ANOVA) and complemented by Dunnett's post hoc test where appropriate. *P *< 0.05 was considered as indicative of significance using GraphPad Software, Prism V. 4.0.

## Results

### *P. elisabethae *anti-inflammatory activity *in vivo *evaluation

The TPA-induced ear oedema model is a classical experimental model of acute inflammation which allows evaluating the anti-inflammatory properties of extracts, fractions and pure compounds, as well studying the presence of several anti-inflammatory mediators at the site of the inflammation. In the present work, we determined, using the mentioned model, the effect of extracts and fractions of *P. elisabethae *(*vide infra*) on important events related to the topical inflammatory process i.e. oedema formation, and MPO release to the oedema tissues.

#### Topical anti-inflammatory activity and tissue MPO assay

**Figure 2:**
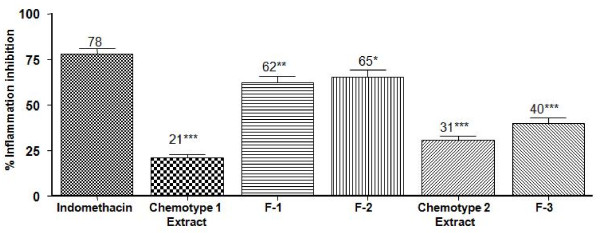
**Effects of extracts and fractions (0.5 mg/ear) from *P. elisabethae *with respect to vehicle (acetone), on the TPA-induced mouse ear oedema**. Data expressed as mean ± S.M.E., *n = 10 *(Anova post-test Dunnet: *P < 0.05, **P < 0.01 and ***P < 0.001 respect to indomethacin (0.5 mg/ear)).

The evaluation of the anti-inflammatory properties of extracts (chemotype 1 and chemotype 2) and fractions, F-1 containing pseudopterosins (PsQ, PsS and PsU), F-2 containing amphilectosins A and B, pseudopterosins (PsG, PsK, PsP and PsT) and *seco-*pseudopterosins (*seco*-PsJ and *seco*-PsK) and F-3 containing non-glycosylated diterpenes (elisabethatrienol, IMNGD, amphilecta-8(13),11,14-triene-9,10-dione and other minor diterpenes), isolated from *P. elisabethae *using the TPA-induced mouse ear oedema is shown in Figure 2. As can be seen in this figure, topical application on the mouse ear oedema of both extracts showed relatively low levels inflammation inhibition of 21 ± 2%, and 31 ± 2%, respectively, when compared to the activity showed by the anti-inflammatory commercial drug indomethacin (78 ± 3%), used as reference in this assay. In contrast, F-1, F-2 and F-3 exhibited inhibition levels of 62 ± 3%, 65 ± 4% and 40 ± 3%, respectively, on the TPA-induced oedema, comparable to that shown by the indomethacin (78 ± 3%).

**Figure 3:**
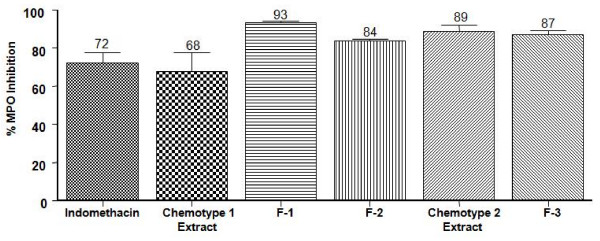
**Effects of extracts and fractions from *P. elisabethae *with respect to vehicle on MPO levels in supernatants of homogenates from TPA-treated ears**. Data expressed as mean ± S.E.M., *n = 3 *(Anova post-test Dunnet: P > 0.05 respect to indomethacin).

MPO assay may be used as an indirect marker of PMNs activation at the site of the inflammatory process [28]. In this context, we analyzed the effect of TPA and concomitant applications and F-1, F-2 and F-3 fractions on the activity of MPO in the mouse ear oedema exudates (Figure 3), after a 4 h treatment. In these experiments we found a marked inhibition of the enzyme activity by the two extracts and fractions tested, indicating high inhibition of neutrophil migration to the site of inflammatory, superior to the inhibition shown by the indomethacin (72 ± 6%).

### *P. elisabethae *anti-inflammatory activity *in vitro *evaluation

An important aspect that must be taken into account in the screening of anti-inflammatory compounds concerns the *in vitro *assays utilized and the prediction of the efficacy of test compounds *in vivo *in order to define their possible clinical relevance. The inflammation is a complex process characterized by the contribution of several mediators including MPO and NO [19,21,29]. In the present work we determined the effect of IMNGD and pure compounds from *P. elisabethae *on the inhibition of MPO released in human PMNs and the NO production in J774 macrophages and also the NO scavenger activity.

#### Inhibition of MPO released by activated human PMNs

The neutrophil granulocyte is a central component of the inflammation process, and has the ability to migrate to the inflammation site and to release toxic products capable of killing invading pathogens. Among the mentioned toxic products, MPO enzyme system is considered to be part of an important antimicrobial system [19] released into the phagosome during the neutrophil degranulation. Thus, we measured the activity of IMNGD and pure compounds from *P. elisabethae *on the release of MPO enzyme on human PMNs using *in vitro *experiments as well. Previously, (data not shown) we established experimentally through MTT cytotoxicity assay that cell viability of human PMNs (>97%) was affected by neither the pure compounds used (up to 10 μM) nor by IMNGD (up to 50 μg/ml) evaluated.

**Figure 4:**
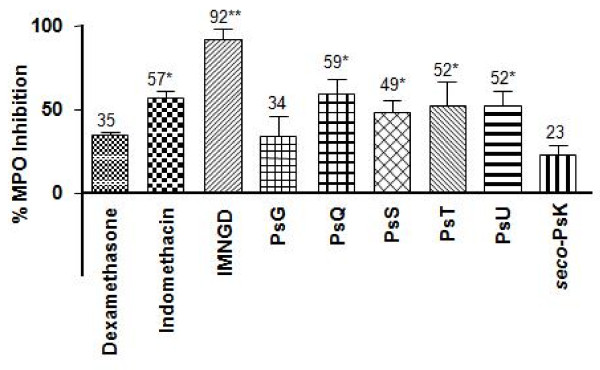
**Effects of pure compounds from *P. elisabethae *on MPO release in PMNs**. Calcium ionophore (1 μM) (A23187)-PMNs (2.5 × 10^6 ^cells/well) treated with IMNGD (50 μg/ml), pure compounds (10 μM), dexamethasone (10 μM) and indomethacin (10 μM). Data expressed as mean ± S.E.M., *n = 3 *(Anova post-test Dunnet: *P < 0.05 and **P < 0.01 respect to control (stimulated cells).

Figure 4 shows the percentage of MPO inhibition (released *in vitro *by human PMNs) by IMNGD (10 μg/ml) and pure compounds (10 μM) isolated from *P. elisabethae*. IMNGD was the most active treatment in the experiment, exhibiting 92 ± 6% inhibition levels in comparison with indomethacin (57 ± 4%) and dexamethasone (35 ± 1%).

In this assay (Figure 4), PsQ (59 ± 4%), PsS (49 ± 4%), PsT (52 ± 4%) and PsU (52 ± 4%) showed similar activity compared to the reference drug indomethacin (57 ± 4%). PsG and *seco*-PsK showed moderate activity, 34 ± 12% and 23 ± 5%, respectively. PsK and PsP were the only pseudopterosins that did not display any activity in this model.

#### Inhibition of NO released in J774 macrophages

Nitric oxide has been shown to have the ability to stimulate COX-2 showing a potential synergism [30]. Nitric oxide appears to be of crucial importance and, this may be considered as a rewarding target for intervention. With this perspective, the present experiment was designed in murine macrophage cells to investigate whether the IMNGD and pure compounds isolated from *P. elisabethae *have any effect on NO production to combat the inflammatory challenge, which is the possible mechanism underlying such an effect. The total NO production in J774 macrophages is an indicator of NO synthesis, an event that occurs during the inflammation process.

In preliminary experiments (data not shown) we established that cell viability (>95%) was affected by neither the pure compounds used (up to 10 μM) nor by the IMNGD (up to 25 μg/ml).

**Figure 5:**
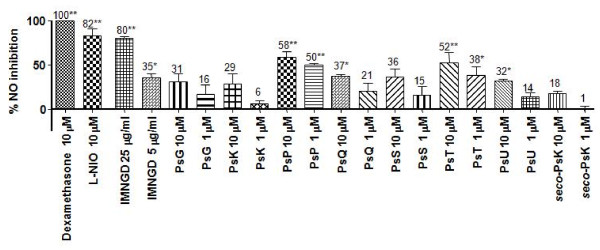
**Effects of pure compounds from *P. elisabethae *on NO release by LPS-stimulated J774 macrophages**. Data expressed as mean ± S.E.M., *n = 3*. (Anova post-test Dunnet: *P < 0.05 and **P < 0.01 respect to control (stimulated cells).

Figure 5 shows the inhibition of NO released in murine macrophages (J-774 cell line) exerted by the IMNGD and pure compounds isolated from *P. elisabethae*. IMNGD at 5 μg/ml had moderate activity (35 ± 5%), but at 25 μg/ml it exhibited an excellent activity with inhibition levels close to 80%. PsP and PsT were the most potent treatments exhibiting inhibition levels between 58–52% at 10 μM and 50–38% at 1 μM, respectively. PsG, PsK, PsQ, PsS, PsU and *seco*-PsK showed low activity (aprox. 25%) even at the highest concentration (10 μM).

#### NO scavenger activity

NO, by inhibiting the generation of pro-inflammatory lipids, exerts anti-inflammatory effects. However, the simultaneous and sustained production of NO and O_2_^- ^leads to the production of toxic species in certain environment, and may cause the reversal of NO effects from protective to deleterious [31]. Thus, the scavenger activity of reactive nitrogen species seems to be important in determining the anti-inflammatory or inflammatory role for NO. In the present experiment, the scavenger effect of IMNGD and pure compounds from *P. elisabethae *on NO was investigated.

**Figure 6:**
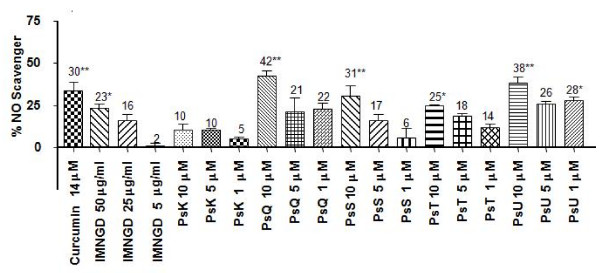
**NO scavenger activity with respect to control showed by pure compounds from *P. elisabethae***. Data expressed as mean ± S.E.M., *n = 3*. (Anova post-test Dunnet: *P < 0.05 and **P < 0.01 respect to control (maximum NO release).

Figure 6 shows the scavenger activity of IMNGD and pure compounds isolated from *P. elisabethae*. The PsQ, PsS and PsU at concentration of 10 μM, exhibited potential NO scavenger percentage of 42 ± 3%, 31 ± 6% and 38 ± 4%, respectively. In contrast, IMNGD, PsK and PsT showed scavenger activity as low as 25%. PsG, PsP and *seco*-PsK did not have any scavenger activity.

## Discussion

The results of the present paper clearly indicates that topical application of the extracts (chemotype 1 and chemotype 2) and fractions F-1 (mixture of pseudopterosins) F-2 (mixture of pseudopterosins, *seco*-pseudopterosins and amphilectosins) and F-3 (mixture non-glycosylated diterpenes) isolated from *P. elisabethae *(Figure 1), and the anti-inflammatory drug indomethacin resulted in a significant inhibition of two important events related to the topical inflammatory response induced by TPA, oedema formation, and PMNs infiltration and degranulation, events that modulate MPO levels at inflammation site. Therefore, these results consistently support that the compounds present in the mentioned fractions possess excellent topical anti-inflammatory properties, similar to as was previously reported for other pseudopterosins as PsA-D [1] and PsM, PsN and PsO [5].

The MPO decrease level even down to basal levels (Figure 3) confirms that the compounds present in the assayed extracts and fractions can reduce the leukocyte infiltration. This was verified on ear homogenates. Based on these results we evaluated *in vitro *whether the pure pseudopterosins and *seco*-pseudopterosin (isolated from F-1, F-2) and IMNGD (isolated from F-3) could have inhibition actions on cellular functions in human PMNs.

Initially, we evaluated leukocyte degranulation of calcium ionophore A23187 stimulated cells (Figure 4). The biomarker used was MPO since this is a recognized granular enzyme engaged in events of activation of PMNs and is associated with tissue injury. Therefore, this is necessary to form the strong oxidant hypoclorous acid, which by reaction with superoxide can in turn generate the reactive hydroxyl radical. In these assays PsQ, PsS, PsT and PsU inhibited significantly the release of MPO in a similar way as the positive controls indomethacin and dexamethasone. In contrast, the IMNGD showed superior inhibition as compared to the positive controls suggesting that glycosylated conditions could reduce the inhibitory activity of these molecules. These results confirm the potential of these molecules and the possibility that they contribute to the inhibition of neutrophil-mediated tissue injury.

Additionally, by comparing the different MPO inhibition values (Figure 4) for the tested compounds in terms of chemical structure, interesting structure-activity relationships arise. First of all, the comparison of the activity of pseudopterosins with different sugar moiety linked to diterpene may indicate that activity depends on: 1) kind of sugar moiety, 2) whether sugar moiety is in a free form or acetylated, 3) acetylation position within the sugar moiety and 4) glycosylation position. For example, PsT glycosylated with non-acetylated arabinopyranose has more activity than PsP which is glycosylated with non-acetylated fucopyranose. Likewise, PsQ and PsS (acetylated fucose as sugar moiety) have more activity than PsP. With regards to the acetylation position, the results showed that acetylation in C-4' of fucose moiety could improve the activity – MPO inhibition value of PsQ (acetylated in C-4') compared to that shown by PsS (acetylated in C-2').On the other hand the glycosylation position might affect the inhibitory activity profile. For example, all pseudopterosins glycosylated in C-10 (PsQ, PsS, PsT and PsU), except PsP, showed more activity than PsG and PsK which are glycosylated in C-9. In the same way, the stereochemistry could be a determinant factor in the inhibition of MPO and leukocyte degranulation, since the activity of PsG and PsK, both glycosylated with fucopyranose but with different stereochemistry in the aglycone (Figure 1), showed different activity. More experiments in relation to this theme should be done to confirm the above discussion.

Regarding NO release in J-774 cell-based assay (Figure 5), we found that the activity of IMNGD and all pure compounds is concentration-dependent. Additionally, IMNGD showed a major activity than the pseudopterosins and *seco*-pseudopterosin. Again as in the MPO assay, the non-glycosylation improves the inhibition of NO release.

By comparing the different NO inhibition values for tested compounds (Figure 5), we also observed structure-activity relationships as with the MPO assay. In general, in this assay the inhibitory activity apparently depends on the glycosylation position (i. e. activity of PsP versus PsG). As to the stereochemistry of the aglycone, it does not seem to be a determinant factor to improve the inhibition (i. e. activity of PsG versus PsK). In contrast the skeleton type might influence the activity. For example, the amphilectane skeleton (PsP) has more inhibitory activity than the serrulatane skeleton (*seco*-PsK). As was mentioned before, more experiments have to be performed to support structure-activity relationships among these kinds of compounds.

In aiming to understanding the behavior of these compounds with respect to their potential as inhibitors of the NO release, we carried out NO scavenger activity assay (Figure 6) to determine whether the inhibition of NO liberation within J-774 cells is produced by inhibition of some molecular process in the cellular machinery (such us inhibition of expression and activity of Inducible Nitric Oxide Synthase (iNOS)), or whether the inhibition is due to scavenger activity [21]. According to the results of these assays, PsG, PsP and *seco*-PsK did not exhibit any scavenger activity, suggesting the possibility that these compounds may inhibit iNOS or other routes that influence this enzyme.

PsQ, PsS, and PsU showed scavenger activity (Figure 6) which let us to confirm that these compounds inhibit NO release in macrophage cells by scavenger activity. However, it is important to carry out more studies in order to confirm if these compounds might inhibit some molecular routes upstream from NO production in cells.

## Conclusion

The results presented here demonstrate that the PsP, PsQ, PsS, PsT and PsU isolated from chemotype 1 and the IMNGD isolated from chemotype 2 are promising molecules with an interesting anti-inflammatory activity profile similar to other compounds of this kind previously described. Additionally, all results confirm that *P. elisabethae *colleted at Providencia and San Andrés Islands has great value as a source of lead compounds with anti-inflammatory properties.

## Competing interests

The authors declare that they have no competing interests.

## Authors' contributions

HC and CD carried out all the procedure for collecting samples, and for the isolation and structure determination of compounds from *P. elisabethae*, while ALV and HC carried out the pharmacological studies and statistical analyses. CD and LFO conceived the study, and participated in its design and coordination. All authors drafted, read and approved the final manuscript.

## References

[B1] Look SA, Fenical W, Jacobs RS, Clardy J (1986). The pseudopterosins: anti-inflammatory and analgesic natural products from the sea whip *Pseudopterogorgia elisabethae*. Proc Natl Acad Sci USA.

[B2] Look SA, Fenical W (1987). The *seco*-pseudopterosins: new anti-inflammatory diterpene-glycosides from a Caribbean gorgonian octocoral of the genus *Pseudopterogorgia*. Tetrahedron.

[B3] Potts BC, Faulkner DJ (1992). Phospholipase A_2 _inhibitors from marine organisms. J Nat Prod.

[B4] Mayer AMS, Jacobson PB, Fenical W, Jacobs RS, Glaser KB (1998). Pharmacological characterization of the pseudopterosins: novel anti-inflammatory natural products isolated from the Caribbean soft coral, *Pseudopterogorgia elisabethae*. Life Sci.

[B5] Ata A, Kerr RG, Moya CE, Jacobs RS (2003). Identification of anti-inflammatory diterpenes from the marine gorgonian *Pseudopterogorgia elisabethae*. Tetrahedron.

[B6] Rodríguez II, Shi Y-P, García OJ, Rodríguez AD, Mayer AMS, Sánchez JA, Ortega E, González J (2004). New pseudopterosin and *seco- *pseudopterosin diterpene glycosides from two Colombian isolates of *Pseudopterogorgia elisabethae *and their diverse biological activities. J Nat Prod.

[B7] Heckrodt TJ, Mulzer J, Mulzer J (2005). Marine Natural Products from *Pseudopterogorgia elisabethae *: Structures, Biosynthesis, Pharmacology, and Total Synthesis. Natural Products Synthesis II.

[B8] Fenical W (1987). Marine soft corals of the genus *Pseudopterogorgia *: a resource for novel anti-inflammatory diterpenoids. J Nat Prod.

[B9] Kijoa A, Sawanwong P (2004). Drugs and cosmetics from the sea. Mar Drugs.

[B10] Gross H, König GM (2006). Terpenoids from marine organisms: unique structures and their pharmacological potential. Phytochem Rev.

[B11] Haefner B (2003). Drugs from the deep: marine natural products as drug candidates. DDT.

[B12] Roussis V, Wu Z, Fenical W, Strobel SA, Van Duyne D, Clardy J (1990). New anti-inflammatory pseudopterosins from the marine octocoral *Pseudopterogorgia elisabethae*. J Org Chem.

[B13] Ata A, Win HY, Holt D, Holloway P, Segstro EP, Jayatilake GS (2004). New antibacterial diterpenes from *Pseudopterogorgia elisabethae*. Helv Chim Acta.

[B14] Ferns TA, Kerr RG (2005). Identification of amphilectosins as key intermediates in pseudopterosin biosynthesis. J Org Chem.

[B15] Puyana M, Narvaez G, Paz A, Osorno O, Duque C (2004). Pseudopterosin content variability of the purple sea whip *Pseudopterogorgia elisabethae *at the Islands of San Andrés and Providencia (SW Caribbean). J Chem Ecol.

[B16] Duque C, Puyana M, Narvaez G, Paz A, Osorno O, Hara N, Fujimoto Y (2004). Pseudopterosins P-V, new compounds from the gorgonian octocoral *Pseudopterogorgia elisabethae *from Providencia Island, Colombian Caribbean. Tetrahedron.

[B17] Duque C, Puyana M, Castellanos L, Arias A, Correa H, Osorno O, Asai T, Hara N, Fujimoto Y (2006). Further studies on the constituents of gorgonian octocoral *Pseudopterogorgia elisabethae *collected in San Andrés and Providencia Islands, Colombian Caribbean: isolation of a putative biosyntetic intermediate leading to erogorgiane. Tetrahedron.

[B18] De Young LM, Kheifets JB, Ballaron SJ, Young JM (1989). Edema and cell infiltration in the phorbol ester-treated mouse ear are temporally separate and can be differentially modulated by pharmacologic agents. Agents Actions.

[B19] Klebanoff SJ (2005). Myeloperoxidase: friend and foe. J Leuk Bio.

[B20] Witko V, Rieu P, Descamps B, Lesavre P, Halbwachs L (2000). Neutrophils: molecules, functions and pathophysiological aspects. Lab Invest.

[B21] Bogdan C (2001). Nitric oxide and the immune response. Nature Immunology.

[B22] National Research Council (1996). Guide for the Care and Use of Laboratory Animals.

[B23] Ministerio de Salud (1993). Resolución 008430. Normas científicas, técnicas y administrativas para la investigación en salud.

[B24] Milano S, Arcoleo F, Dieli MD, Agostino RD, Agostino P, De Nucci G, Cillari E (1995). Prostaglandin E2 regulates inducible nitric oxide synthase in the murine macrophage cell line J774. Prostaglandins.

[B25] Bradley PP, Priebat DA, Christensen RD, Rothstein G (1982). Measurement of cutaneous inflammation: estimation of neutrophil content with an enzyme marker. J Invest Derm.

[B26] CYTED (2002). Técnicas *in vitro *para el estudio de fármacos antiinflamatorios. Subprograma X. Proyecto X.6. Búsqueda y evaluación de nuevos agentes naturales con actividad antiinflamatoria y antiartrítica.

[B27] Marcocci L, Magguire JJ, Droy-Lefaix MT, Packer L (1994). The nitric oxide-scavenger properties of *Ginkgo biloba *extract EGB 761. Biochem Biophys Res Commun.

[B28] Frode TS, Medeiros YS (2001). Myeloperoxidase and adenosine-deaminase levels in the pleural fluid leakage induced by carrageenan in the mouse model of pleurisy. Mediators Inflammation.

[B29] Vliet A Van der, Eiserich JP, Cross CE (2000). Nitric oxide: a proinflammatory mediator in lung disease?. Respir Res.

[B30] Hughes FJ, Buttery LD, Hukkanen MV, O'Donnell A, Maclouf J, Polak JM (1999). Cytokine induced prostaglandin E2 synthesis and cyclooxygenase-2 activity are regulated both by a nitric oxide dependent and independent mechanism in rat osteoblasts *in vitro*. J Biol Chem.

[B31] Grisham MB, Jourd'Heuile D, Wink A (1999). Nitric Oxide I: Physiological chemistry of nitric oxide and its metabolites: implications in inflammation. Am J Physiol Gastrointest Liver Physiol.

